# One-shot exogenous interventions increase subsequent coordination in Denmark, Spain and Ghana

**DOI:** 10.1371/journal.pone.0187840

**Published:** 2017-11-16

**Authors:** Anna Lou Abatayo, Bo Jellesmark Thorsen

**Affiliations:** 1 Department of Economics, Bocconi University, Milan, Italy; 2 Fondazione Eni Enrico Mattei, Milan, Italy; 3 Department of Food and Resource Economics, University of Copenhagen, Copenhagen, Denmark; 4 Center for Macroecology, Evolution and Climate, University of Copenhagen, Copenhagen, Denmark; Middlesex University, UNITED KINGDOM

## Abstract

Everyday, we are bombarded with periodic, exogenous appeals and instructions on how to behave. How do these appeals and instructions affect subsequent coordination? Using experimental methods, we investigate how a one-time exogenous instruction affects subsequent coordination among individuals in a lab. Participants play a minimum effort game repeated 5 times under fixed matching with a one-time behavioral instruction in either the first or second round. Since coordination behavior may vary across countries, we run experiments in Denmark, Spain and Ghana, and map cross-country rankings in coordination with known national measures of fractualization, uncertainty avoidance and long-term orientation. Our results show that exogenous interventions increase subsequent coordination, with earlier interventions yielding better coordination than later interventions. We also find that cross-country rankings in coordination map with published national measures of fractualization, uncertainty avoidance, and long-term orientation.

## Introduction

Everyday, we are bombarded with periodic, exogenous appeals and instructions on how to behave. We are reminded to vaccinate our children and keep them away from daycare and school when they are sick, we are told what behavior is acceptable when utilizing shared office facilities (e.g., cleaning up after yourself when you use the office kitchen), and we are instructed how to keep our neighborhoods clean (e.g., no littering, staying off the grass, and picking up after your dog). These information campaigns and reminders of expected conduct are important, insofar as they serve as coordination devices, especially when, at the most basic level, individuals are unable to coordinate well [1–3].

An explanation for coordination failure could be that individuals have diffused prior beliefs regarding the coordination outcome. In a standard minimum effort game, an individual’s effort choice is highly dependent on the effort choice he expects from his group members. Given his expectations, an individual will first act on his home-grown beliefs about what is appropriate and then update these beliefs as he observes and interacts with others. Hence, any information or experience that affects his expectations will affect his behavior. We conjecture that exogenous appeals and instructions on how to behave, similar to those mentioned above, do just that.

In this paper, we experimentally investigate how exogenous appeals and instructions (henceforth, exogenous interventions) affect an individual’s subsequent coordination behavior. In a between subjects design with fixed matching, we have participants play the standard minimum effort game for 5 rounds. In one treatment, which we call the R1Int Treatment, participants are told, after first round instructions, that they **MUST** pick the Pareto dominant effort level for that round. In another treatment, which we call the R2Int Treatment, participants are told, after the second round instructions, that they **MUST** pick the Pareto dominant effort level for that round. We compare post-intervention coordination behavior of individuals in our treatments with those who were not told to pick an effort level (NoInt Treatment).

We run separate experiments among a group of Danes, a group of Spaniards, and a group of Ghanaians. We do this for several reasons. First, general experimental literature shows that there may be considerable cross-country variation in individual behavior in terms of cooperation, coordination and learning [4–10]. Our experimental design aims to document these differences and offer some observable correlations in the discussion. Second, in the more specific experimental literature on coordination, only one paper—to our knowledge—has looked at cross-country effects. In this paper Engelmann and Normann [9] compare their experiments in Denmark with similar experiments on students run by others. Their results show that Danes are able to coordinate better than non-Danes. From their own experiments, they also find that when Danes are in a group with non-Danes, the proportion of Danes in the group can be used to predict how well-coordinated the group will be. However, since the experiments were run by different researchers and used for different research questions, cross-country differences in the behavior of Danes and non-Danes in Engelmann and Norman [9] may possibly be confounded by experimental differences. Our design provides a cleaner way of studying cross-country differences. Lastly, while previous experimental results using a Danish sample drove our decision to run experiments in Denmark, our choice of Spain and Ghana were largely driven by the desire to exploit as much cross-country variation while keeping the time zones—and hence, the experiment times—across countries constant. Our desire to minimize as much cross-country variation as possible led to our choice of participants (university students), solicitation method (pen-and-paper), instructor nationality (all locals but all trained together in Denmark), experimental money to real money exchange rates (purchasing power parity converted and relative to the country’s minimum wage), and of course, time and season (experiments were simultaneously run in spring). Running simultaneous experiments in Denmark, Spain and Ghana was also made easier by the fact that logistics in these countries had already been set for other experiments that required participant interaction across countries.

We note that although there is literature claiming that students behave similarly to the general population [11–13], we do not claim our student population to be representative of the population in different countries. They do, however, share history and culture with the rest of their populations. With that caution in mind, we note that ethnic, linguistic and religious fractualization measures published by Alesina, et al. [14] are highest in Ghana and higher in Spain than in Denmark. These imply that Danes are more homogeneous, and hence, possibly better at coordinating than Ghanaians and Spaniards.

Geert Hostede measures of uncertainty avoidance and long-term orientation may also be correlated with coordination behavior [15]. On one hand, “uncertainty avoidance” measures the extent to which individuals of a culture feel threatened by unknown situations; and hence, can proxy for our participants’ level of ambiguity aversion. We expect individuals from countries with high uncertainty avoidance to coordinate closer to the risk dominant equilibrium. Our reason for this is as follows: individuals who are ambiguity averse will be averse to what in the literature is called social uncertainty (also referred to as strategic uncertainty), the uncertainty that arises because a participant is unsure of what other individuals in his or her group will choose [16, 17]. Hence, an ambiguity averse participant will pick levels closer to the risk dominant equilibrium as these levels insure him from losses associated with picking a level higher than the minimum choice in the group. On the other hand, “long-term orientation” measures how each society maintains its link to the past while dealing with present and future challenges. Countries that score low are those that are very traditional, whose citizens are likely to hold on to past grudges and more likely to view societal change with suspicion. Long-term orientation measures, then, can proxy for how likely an intervention will affect a participant’s subsequent behavior. Interventions can be viewed as a new way of doing something. Participants who have high long-term orientation measures are more likely to be open to the experience derived from the intervention and hence, more likely to have better post-intervention coordination experiences. Among our three countries, Spain has the highest uncertainty avoidance and long-term orientation measure, Denmark has the lowest uncertainty avoidance measure, while Ghana has the lowest long-term orientation measure.

Our results show that, indeed, under a no intervention baseline, Danes are able to coordinate better than Spaniards and Ghanaians. While initial coordination in Spain and Ghana are similar, we find that the level at which Spaniards coordinate decline faster than the level at which Ghanaians coordinate, leading us to conjecture that our Spanish participants are more ambiguity averse. These results are correlated with measures of fractualization and uncertainty avoidance.

We also find that although individuals are not required to comply with the exogenous intervention, all individuals actually do comply. Hence, all individuals experience how it is when they are able to coordinate at the highest possible level. A round after the intervention, coordination in all 3 countries continue to be higher than baseline, but only when the intervention happens in the first round. Two and three rounds after a first-round intervention, coordination among Spaniards and Danes continue to be higher than baseline. When intervention happens in the second round, Spaniards coordinate better than baseline a round after the intervention, but not two and three rounds after. The same result, however, does not hold for Danes and Ghanaians. An intervention in the second round does not lead to changes in the coordination behavior among Danes and Ghanaians. We suggest that these results could be driven by differences in long-term orientation. Greater openness to the intervention experience, i.e. high long-term orientation, leads to more effective intervention results.

## Related literature

There is extensive experimental literature on coordination games, many of which investigate how better coordination among individuals can be achieved. A review of several articles on coordination games since 1992 reveals that coordination among individuals increase with communication, repetition, smaller group sizes, lower risk, and the observability of other people’s actions [18]. On top of this, many authors have also found that financial incentives [19–21], gradually increasing the upper bound of a coordination game [22], the number of males in a group [23], positive spillovers [24], and truth-telling oaths [25] increase coordination. In terms of financial incentives, Fehr and Tyran [26] find that real payoffs yield better coordination than nominal payoffs. There is also discussion on whether non-monetary sanctions and rewards are just as effective in fostering coordination as financial incentives [21, 27].

What is perhaps more relevant and closely tied to our study is the effect of information, advice and recommendations on coordination behavior. Information affects individual expectations which, in turn, affect individual choices [2]. Revealing each player’s individual strategy as opposed to just revealing the group’s minimum choice, for example, has been found to increase coordination [28, 29]. Being given information regarding the play of a group member who picks the Pareto dominant equilibrium also increases coordination [30]. In another version of a coordination game called the *dying seminar*, Semeshenko et al. [31] examine what happens to coordination when information is progressively decreased. They find that knowing the actual number of participants in the previous round is enough for participants to coordinate at the Pareto optimal equilibrium.

The effect of advice and recommendations on coordination behavior is very similar to the effect of information. In fact, an additional piece of advice or recommendation can be considered an additional information given to experiment participants. But while information in the context above comes from within (i.e., providing participants with knowledge on how coordinated their group is and what the other members of the group decided on in the previous round), advice and recommendations can come from a third party and may not necessarily be about the group and its members. Chaudhuri et al. [32] investigate the effect of an advice in a non-overlapping generations coordination game and find that coordination among participants is better when advice is made public and distributed in a way that makes it common knowledge. Making recommendations common knowledge for all members of the group is also a key ingredient for recommendations to successfully resolve coordination failure in a coordination game with fixed matching [21].

The role of information, advice and recommendations has also been looked at outside the coordination literature. Many have looked at the effect of peer information on pro-social behavior as a way to increase contributions to public goods provision [33–37], conservation [38–41] and charitable giving [42–45]. There are also those who have looked at the role of intergenerational advice on public goods provision [46, 47], trust and generosity [48] and resource extraction [49]. These papers find that positive information or advice increases pro-social behavior while negative information or advice deters it.

Although we draw insights from the literature outside coordination, our paper is primarily about coordination and as such, contributes to the existing knowledge on how better coordination can be achieved. In particular, we investigate, in three different countries, how an exogenous intervention that recommends a particular behavior can positively lead to better future coordination. We conjecture that this kind of positive intervention can update an individual’s posterior expectations of others’ behavior and change their subsequent coordination behavior, with the magnitude of the effect differing across countries.

To a certain extent, our intervention can be thought of as a top-down establishment of a social norm. Our intervention creates a focal solution to a coordination problem and hence reduces the risk of coordination [50]. Moreover, since our intervention is costless and enforcement is non-existent, our intervention can also be thought of as a way of nudging individuals towards the Pareto dominant equilibrium [51]. However, our results show that although subsequent coordination is higher after an intervention, subsequent declines in coordination suggest that the intervention has failed to become a social norm. Our intervention is also too paternalistic to truly be considered a nudge.

Given these, our contribution to the coordination literature is two-fold. First, to our knowledge, our paper is the first to study how a one-time exogenous intervention in the form of a behavioral instruction affects subsequent coordination. Our paper, to our knowledge, is also the first to explore how basic coordination and post-intervention coordination differ across countries.

## Materials and methods

A total of 60 experiment sessions (20 session in each country) were conducted in Denmark, Spain and Ghana from April to May 2016. Participants in Denmark and Spain were recruited using ORSEE [52] while participants in Ghana were recruited using flyers and in-class advertisements. As in Denmark and Spain, participants in Ghana were randomly invited to experimental sessions and as such, could not self-select into treatments. Most of our participants were students at the University of Copenhagen, Pompeu Fabra University, and University of Ghana, where the experiments were conducted. (Two individuals in Denmark, six individuals in Spain, and three individuals in Ghana were no longer students at the time of our experiment. All these individuals had already obtained their undergraduate degrees.) All were national citizens of the country they were taking the experiment in.

In accordance with Danish legislation, our study did not require an institutional review board (IRB), as sensitive data, as defined by the Danish Protection Agency, was not retrieved from participants. Participants signed a consent form and were advised that they were free to leave at any time during the experiment. A copy of the participant consent form in English can be found in the supplementary information (S1 File). Participants also signed a payment form when they received payment for the experiment. The complete dataset, without any identifying information regarding our participants, has been posted in FigShare (DOI 10.6084/m9.figshare.5178229).

Instructors and experimenters were trained in Denmark prior to the experiments. All instructors were nationals of the country they instructed experiments in, and only the instructors interacted with experiment participants during the experiment. The only time experimenters interacted with participants was when the latter received their payments, after having completed the experiment.

All of our sessions had 12 participants each, except for 2 sessions in Denmark and 2 sessions in Spain that only had 8 participants. One of these two sessions in Denmark and a different session in Spain had to be dropped from our observations due to experimental error. (The experimenter told everyone to pick the number 4 during a baseline session. We dropped this session from our data.) Hence, we had a total of 684 participants: 220 participants from Denmark, 224 participants from Spain, and 240 participants from Ghana.

Instructions were given using the country’s academic language of instruction: Danish in Denmark, Spanish in Spain and English in Ghana. They were initially written in English, translated to Danish and Spanish by native speakers and re-translated to English by a different set of native speakers [53]. This is to ensure that words and sentences are translated well and that participants are not primed or framed in any way. We pay particular attention to the translation of the word “must”. It is translated to Danish as “skal” and to Spanish as “debe”. Re-translation successfully translated it back to “must”. English instructions from the experiment in Ghana can be found in the supplementary information (S1 File). Spanish and Danish instructions are available upon request.

Average earnings were 147.65 DKK, 12.84 EUR and 10.09 GHS, and sessions ran for 40 minutes on average. The minimum hourly wage in Denmark, Spain and Ghana are estimated to be 110 DKK, 6 EUR and 7 GHS, respectively. All sessions were conducted using pen and paper due to power outages and unreliable internet connections in Ghana. Participants were unable to see other participants’ decision sheets: they were either assigned a table with partitions or were seated at least two seats apart. Communication among participants was disallowed during the experiment. All participants were asked for their consent, and once everyone consented to participating in the experiment, were randomly and anonymously assigned to groups of 4. They remained in the same group for the duration of the experiment and the identities of their group members were never revealed. Instructions were read out loud and participants were asked to answer a few questions to test their understanding of the instructions.

**Fig 1:**
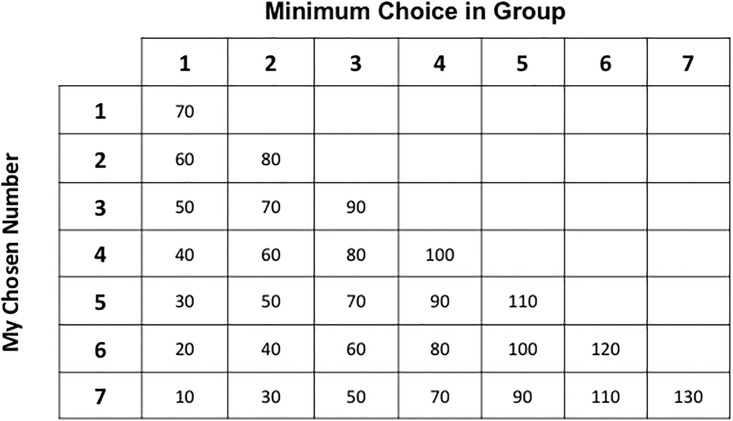
Payoff table for the minimum effort game.

All participants participated in a standard minimum effort game for 5 rounds. They were asked to choose an integer between 1 and 7, inclusive of 1 and 7, and were told that their payoffs depended on their chosen number and the minimum number chosen by all the members of their group. The game has 7 Nash equilibria, where all individuals in the group choose the same effort level. Everyone choosing 7 is the Pareto dominant equilibrium while everyone choosing 1 is the risk dominant equilibrium. Fig 1 shows the payoff table that we used in the experiment. It is standard to the literature and is exactly the same payoff table used in Van Huyck, et al. [2], Cooper, et al. [54], and Engelmann and Normann [9].

Sessions differed in whether participants were told to pick the the Pareto dominant effort level, i.e., pick “7”, in the first round (R1Int), in the second round (R2Int) or not told at all (NoInt). During the round where participants were asked to pick “7”, they were free to deviate from what was asked of them. There was no punishment for deviating. A summary of our experimental design and the number of observations per treatment is found in Table 1.

**Table 1:** Summary of experimental design.

**Denmark**			
**Code**	**Treatment Name**	**# Participants**	**# Ind. Groups**
NoInt	No Intervention	72	18
R1Int	Intervention in Round 1	80	20
R2Int	Intervention in Round 2	72	18
**Spain**			
**Code**	**Treatment Name**	**# Participants**	**# Ind. Groups**
NoInt	No Intervention	72	18
R1Int	Intervention in Round 1	76	19
R2Int	Intervention in Round 2	72	18
**Ghana**			
**Code**	**Treatment Name**	**# Participants**	**# Ind. Groups**
NoInt	No Intervention	84	21
R1Int	Intervention in Round 1	84	21
R2Int	Intervention in Round 2	72	18

*Notes:* Two sessions in Denmark and Spain were dropped due to an experiment error. These sessions belonged to “NoInt”. 1 session in Denmark and 2 sessions in Spain only had 2 groups of 4. All three sessions belonged to “R1Int”. The rest of our sessions had 3 groups of 4.

## Results

Most of our participants were university students in business, sciences, social sciences, humanities and liberal arts. All of them were above 18 years of age. On average, Spanish participants were younger than Ghanaian participants, and Ghanaian participants, in turn, were younger than Danish participants. A majority of our participants in Ghana, almost half of our participants in Denmark, and less than half of our participants in Spain were males. Denmark had the most number of participants who were married and who have at least 1 child. A summary of these demographic characteristics is included in the supplementary information (S1 Appendix).

While we see dissimilar gender proportions across countries, gender proportions across treatments within each country are statistically similar. We also find that, within each country, the age of participants are similarly distributed. The same holds true for civil status and number of children. A summary of these statistically tests is included in the supplementary information (S2 Appendix). These support the fundamental assumption that participants within each country are drawn from the same population of university students, and allow us to compare participant choices across treatments. That is, along with the facts that (1) treatments were assigned to sessions such that participants cannot select into treatments and (2) we have a sufficiently large sample size, we can attribute the behavior of participants in each treatment to treatment differences and not to individual differences.

In the following subsections, we first examine cross-country differences in coordination before looking at the effects of a first-round and a second-round intervention on subsequent coordination. We then compare between interventions and examine whether one intervention yields better subsequent coordination results than the other. We present results from regressions in this paper. Similar results can be obtained using one-tailed t-tests. Results from the one-tailed t-tests can be found in the supplementary information (S3 Appendix). For convenience, we label all rounds after an exogenous intervention as “PostRound”. That is, in R1Int, round 2 is labeled PostRound1, round 3 is labeled PostRound2, etc. Similarly, in R2Int, round 3 is labeled PostRound1, round 4 is labeled PostRound2, etc. Since participants under NoInt are not asked to pick “7” in any of their rounds, round 1 is the same as PostRound1, round 2 is the same as PostRound2, etc.

### Baseline coordination results

**Fig 2:**
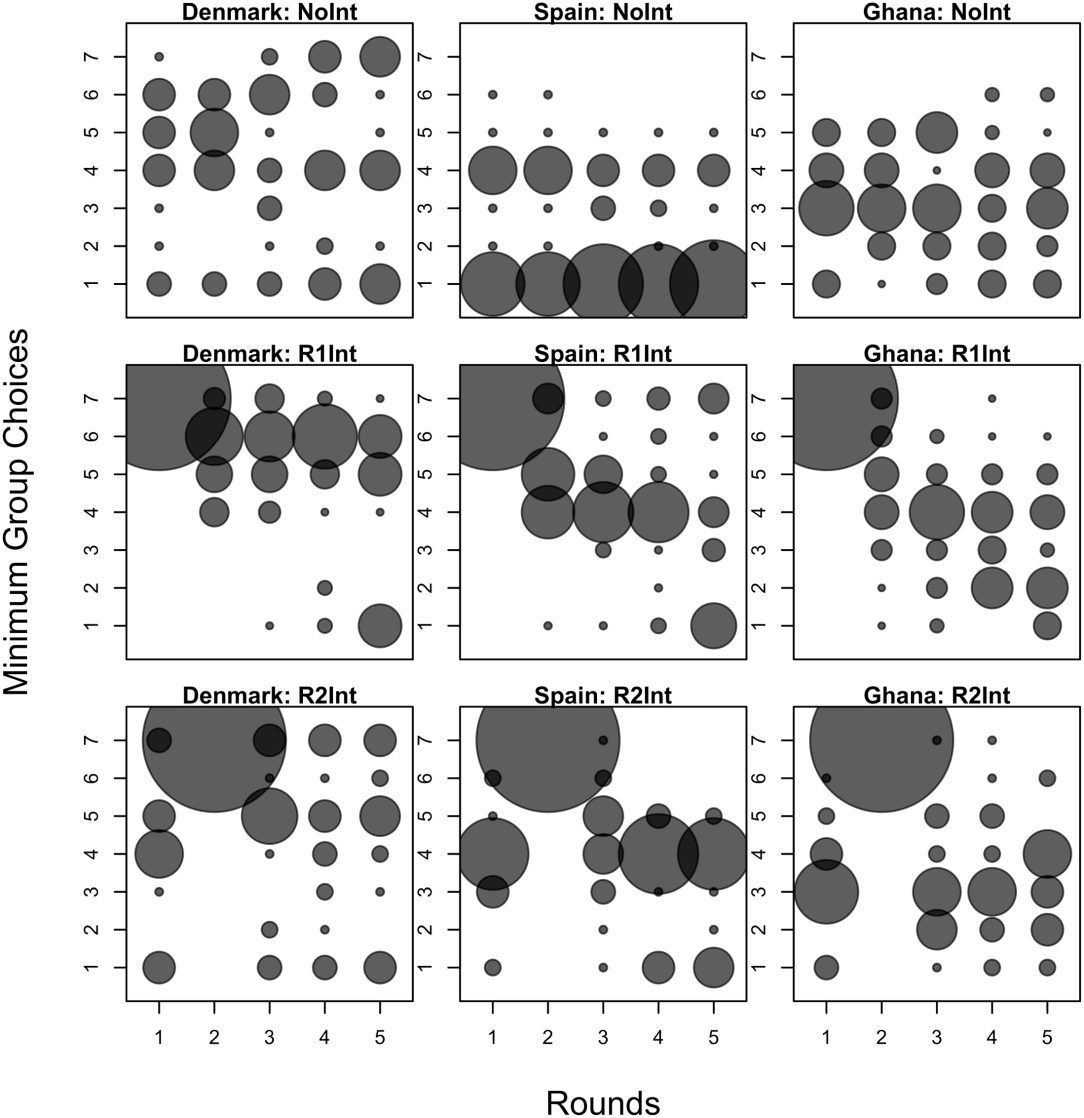
Distribution of minimum group choices. The radii of the circles are calculated based on the percentage of groups with a particular minimum effort level over the total number of groups per country and treatment.

Looking at average contributions under NoInt, we find our results consistent with what has been found in the literature. The less ethnically, linguistically and religiously fractualized Danes are able to coordinate better than their more fractualized counterparts in Spain and Ghana. On average, Spaniards and Ghanaians choose effort levels between 3 and 5 while Danes choose effort levels between 5 and 6. As for how well Danes, Spaniards and Ghanaians are able to coordinate with one another, the top part of Fig 2 shows that Danes are better able to coordinate at higher levels than Spaniards and Ghanaians. Regression results in columns 1 and 5 of Table 2 show that Danes coordinate better than Ghanaians, increasing Danish profits from coordination by 14.60 tokens.

**Table 2:** Baseline coordination results.

Dependent Variable:	Tokens Earned				Minimum Choice in Group			
	All	Denmark	Spain	Ghana	All	Denmark	Spain	Ghana
	(1)	(2)	(3)	(4)	(5)	(6)	(7)	(8)
Denmark	14.6032** (6.3812)				1.0695* (0.5760)			
Spain	-4.1468 (4.6340)				-0.9435** (0.4526)			
PostRound2		3.4722 (3.7319)	1.9444 (2.0683)	1.0714 (5.6239)		0.0608 (0.1826)	0 (0.1343)	0.0506 (0.3871)
PostRound3		5.8333 (5.8379)	9.1667* (4.6791)	-0.5952 (4.4799)		0.0002 (0.2998)	-0.5443** (0.2544)	-0.2004 (0.3421)
PostRound4		8.3333 (7.0642)	10.6944** (4.7178)	2.0238 (4.4655)		0.0181 (0.4399)	-0.5705** (0.2685)	-0.2138 (0.3847)
PostRound5		9.1667 (7.4512)	11.9444** (5.0753)	1.1905 (5.1030)		-0.0345 (0.5128)	-0.7300** (0.3686)	-0.273 (0.4151)
Constant	76.4524*** (3.4542)	85.6944*** (7.0078)	65.5556*** (6.5626)	75.7143*** (4.8231)				
Constant_Cut1					-1.0180*** (0.2645)	-1.3768** (0.5613)	-0.2845 (0.5056)	-1.8464*** (0.5541)
Constant_Cut2					-0.5958** (0.2717)	-1.0592** (0.4728)	-0.1024 (0.4966)	-0.9946** (0.4533)
Constant_Cut3					0.0915 (0.2574)	-0.8368* (0.4277)	0.2803 (0.4942)	0.2892 (0.3567)
Constant_Cut4					1.2630*** (0.2815)	0.1913 (0.3874)	2.1364** (0.8446)	1.2731*** (0.3820)
Constant_Cut5					2.1784*** (0.2831)	0.7573** (0.3847)	3.4546*** (1.0148)	3.1144*** (0.5539)
Constant_Cut6					3.4213*** (0.4041)	1.8839*** (0.4281)		
R2/PseudoR2	0.09	0.01	0.04	0	0.05	0	0.01	0
N	1140	360	360	420	1140	360	360	420

*Notes:* OLS run for columns (1) to (4) and ordered logit run for columns (5) to (8). *Denmark* and *Spain* are dummy variables that take on the value of 1 if a participant is Dane or Spaniard, respectively. *PostRound2*, *PostRound3*, *PostRound4*, and *PostRound5* are PostRound dummies that take on the value of 1 if an observation comes from a particular PostRound. Standard errors clustered on a group level in parentheses.

*** *p* < 0.01,

** *p* < 0.05,

* *p* < 0.10.

**Fig 3:**
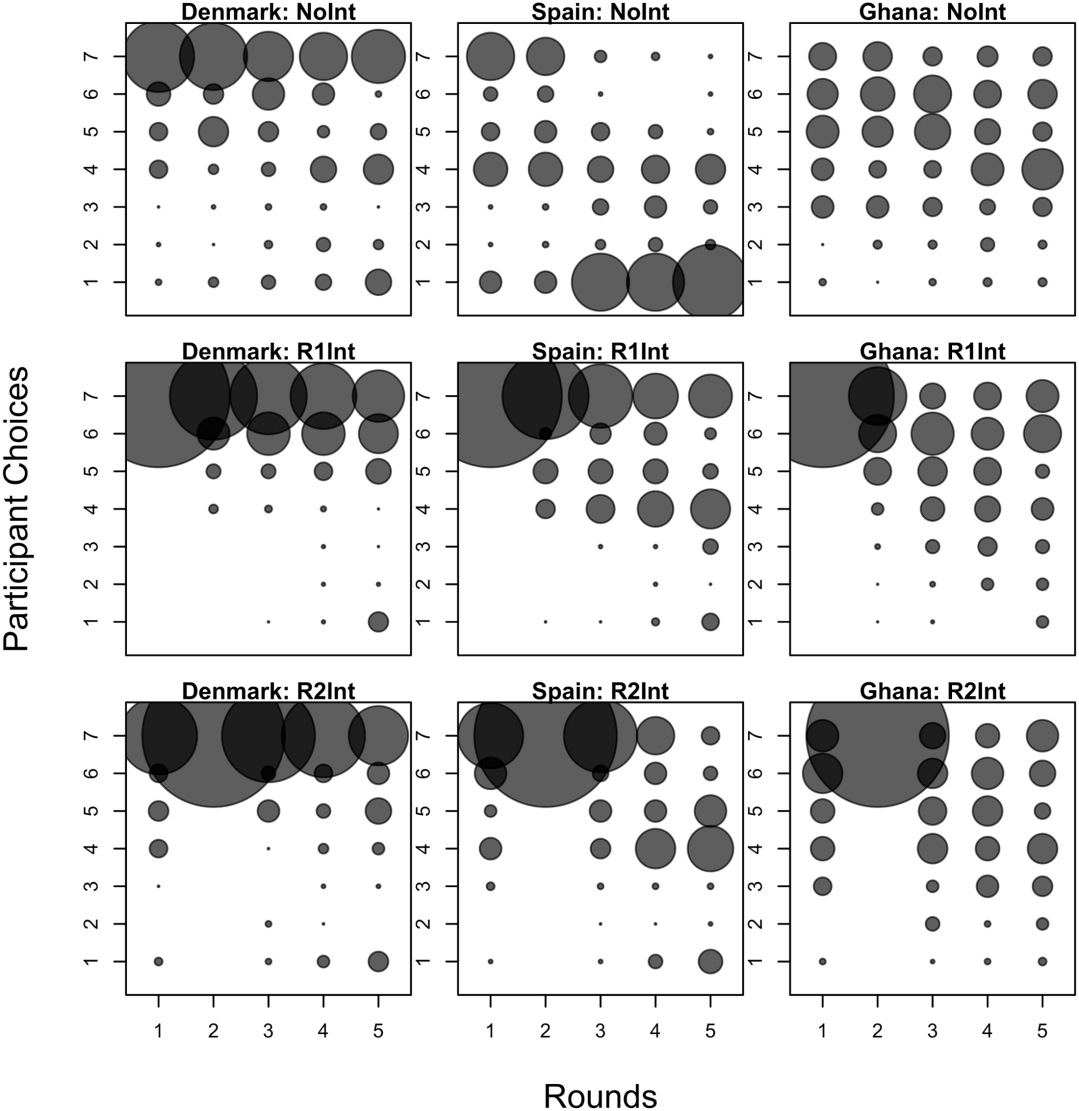
Distribution of participant choices. The radii of the circles are calculated based on the percentage of participants choosing a particular effort level over the total number of participants per country-treatment. The total number of participants per country-treatment is available in Table 1.

Columns 1 and 5 of Table 2 also show that although Spaniards coordinate at levels lower than Ghanaians, this does not translate to lower Spanish profits. In fact, when we look at columns 3 and 7 of Table 2, we find that although the level at which Spaniards coordinate decreases the longer they are in the game, Spanish profits from coordination are increasing. The reason for these two seemingly contradictory results is this: Spaniards are able to coordinate better but at lower levels. Looking at Fig 3, we see an increasing number of Spaniards picking the lowest effort level starting from round 3.

We conjecture that the rapid decline in the level at which Spaniards coordinate is due to Spaniards being more risk-averse than Ghanaians and Danes. While Spaniards initially coordinated at levels similar to Ghanaians, miscoordination drove Spaniards to rapidly decrease their coordination level towards the risk-dominant equilibrium. This result is in line with the fact that Spaniards have higher measures of uncertainty avoidance than Ghanaians. Looking at coordination in the fourth and fifth rounds, we find that Spaniards are unable to recover from this decline.

**Result 1** *Danes are able to coordinate at higher levels than Spaniards or Ghanaians*. *The level at which Spaniards coordinate declines more rapidly than the level at which Danes or Ghanaians coordinate*.

### Intervened coordination results

Despite there being no enforcement and monitoring, all participants picked as instructed during the intervention round. When the intervention happens in the first round, Danes, Spaniards and Ghanaians coordinate at much higher levels the round right after the intervention compared to a similar round under NoInt. In particular, Danes coordinate at an average level of 5.5 (median group coordination: 5) in PostRound1 of R1Int compared to 4.17 (median group coordination: 4) in PostRound1 of NoInt, Spaniards coordinate at an average level of 4.85 (median group coordination: 4) in PostRound1 of R1Int compared to 2.36 (median group coordination: 2) in PostRound1 of NoInt, and Ghanaians coordinate at an average level of 4.36 (median group coordination: 3) in PostRound1 of R1Int compared to 3.19 (median group coordination: 3) in PostRound1 of NoInt. Regression results on tokens earned (see Table 3) and minimum choice in group (see Table 4) show statistically significantly higher coordination and profits from coordination in all three countries. Coordination profits, after a first-round intervention, in PostRound1 increases in Denmark by 20-23 tokens, in Spain by 30 tokens, and in Ghana by 17-19 tokens.

**Table 3:** Intervention results on tokens earned.

Dependent Variable:	Tokens Earned					
	Denmark		Spain		Ghana	
	(1)	(2)	(3)	(4)	(5)	(6)
NoInt * PostRound2	3.4722 (3.6610)	3.4722 (3.6751)	1.9444 (2.0296)	1.9718 (2.0571)	1.0714 (5.5391)	0.9756 (5.6256)
NoInt * PostRound3	5.8333 (5.7270)	5.8333 (5.7490)	9.1667 *(4.5915)	9.2958 *(4.6572)	-0.5952 (4.4124)	-0.4878 (4.5201)
R1Int * PostRound1	20.4306** (7.7103)	22.3500*** (7.3630)	30.2339*** (8.2914)	30.3262*** (8.3763)	17.3810** (7.4870)	18.8069** (7.4924)
R1Int * PostRound2	19.5556** (8.5678)	21.4750*** (7.9168)	25.4971*** (8.0650)	25.7928*** (8.1947)	5.7143 (6.4556)	5.8955 (6.5472)
R1Int * PostRound3	12.6806 (9.3897)	14.6 (8.9417)	28.7865*** (8.4064)	28.9928*** (8.5372)	7.5 (6.1778)	8.174 (6.2162)
R2Int * PostRound1	2.2222 (10.8535)	3.8449 (10.1507)	21.2500** (8.1464)	21.5328** (8.1047)	1.9246 (6.9007)	2.5144 (6.9662)
R2Int * PostRound2	1.9444 (10.2975)	3.8449 (9.7744)	14.4444* (7.7413)	14.7272* (7.7380)	5.2579 (7.1705)	6.485 (7.4083)
R2Int * PostRound3	10.2778 (9.5476)	12.0139 (9.2397)	14.7222* (7.7307)	15.0050* (7.7596)	2.0635 (6.3791)	2.9556 (6.4481)
Gender		6.6292** (3.2837)		-0.0983 (2.7668)		2.8093 (3.0460)
Age		-0.9780** (0.4333)		0.4821 (0.3070)		-0.5993 (0.8516)
Uncooperative		-0.8936 (1.6255)		1.4481 (1.1713)		1.1061 (1.2625)
Trust		0.0243 (1.7785)		-0.3198 (1.2972)		-0.6393 (1.1010)
Risk		2.1008* (1.0488)		0.0641 (0.7090)		0.3097 (0.6309)
Constant	85.6944*** (6.8747)	92.7504*** (16.3276)	65.5556*** (6.4397)	51.5023*** (10.6120)	75.7143*** (4.7503)	84.3420*** (21.9536)
R-squared	0.06	0.11	0.15	0.16	0.05	0.06
N	672	669	660	654	720	687

*Notes:* OLS regressions run. *NoInt*, *R*1*Int* and *R*2*Int* are dummies that take on the value of 1 if an observation belongs to NoInt, R1Int or R2Int, respectively. *PostRound*1, *PostRound*2 and *PostRound*3 are PostRound dummies. All variables with “*” are interaction terms. Gender is 1 when a participant is male. The rest of the variables are treated as continuous. Standard errors clustered on a group level in parentheses.

*** *p* < 0.01,

** *p* < 0.05,

* *p* < 0.10.

**Table 4:** Intervention results on coordination.

Dependent Variable:	Minimum Choice in Group					
	Denmark		Spain		Ghana	
	(1)	(2)	(3)	(4)	(5)	(6)
NoInt * PostRound2	0.0508 (0.2054)	0.0392 (0.2155)	0 (0.1314)	0.0191 (0.1311)	0.052 (0.3838)	0.0458 (0.3869)
NoInt * PostRound3	0.0395 (0.3622)	0.0635 (0.3695)	-0.5589** (0.2593)	-0.5536** (0.2651)	-0.2228 (0.3287)	-0.2232 (0.3347)
R1Int * PostRound1	1.1573** (0.4847)	1.3710*** (0.4726)	2.6093*** (0.6919)	2.6059*** (0.7082)	1.5678*** (0.5839)	1.6275*** (0.5856)
R1Int * PostRound2	1.1965** (0.5273)	1.3923*** (0.5186)	1.9966*** (0.6583)	1.9821*** (0.6783)	0.4294 (0.5283)	0.4405 (0.5351)
R1Int * PostRound3	0.8809 (0.5634)	1.0406* (0.5433)	1.8860** (0.7398)	1.8586** (0.7571)	0.2953 (0.5267)	0.3593 (0.5192)
R2Int * PostRound1	0.3773 (0.6315)	0.4745 (0.6262)	1.8254** (0.7279)	1.8452** (0.7241)	-0.1616 (0.5587)	-0.1411 (0.5621)
R2Int * PostRound2	0.1067 (0.6386)	0.2381 (0.6604)	0.9521 (0.6325)	0.9646 (0.6403)	0.0582 (0.6109)	0.1037 (0.6387)
R2Int * PostRound3	0.3717 (0.6664)	0.5121 (0.6783)	0.5641 (0.6316)	0.5545 (0.6432)	-0.1415 (0.5612)	-0.1404 (0.5794)
Gender		0.5560** (0.2190)		0.1644 (0.2433)		0.1841 (0.2832)
Age		-0.0596** (0.0297)		0.0418 (0.0342)		-0.0832 (0.0632)
Uncooperative		-0.0386 (0.1179)		0.077 (0.1116)		-0.0279 (0.0978)
Trust		0.0165 (0.1155)		0.0609 (0.1222)		-0.0106 (0.0888)
Risk		0.1724** (0.0685)		0.0759 (0.0618)		-0.0027 (0.0498)
Constant_Cut1	-1.4794*** (0.4903)	-1.5201 (1.0151)	-0.3342 (0.4891)	1.4229 (1.0503)	-2.1983*** (0.5353)	-4.0421** (1.7549)
Constant_Cut2	-1.1461*** (0.4419)	-1.1648 (0.9886)	-0.1427 (0.4983)	1.6177 (1.0371)	-0.9202** (0.3925)	-2.8041* (1.6176)
Constant_Cut3	-0.8681** (0.4073)	-0.8696 (0.9795)	0.3025 (0.4910)	2.0707** (1.0320)	0.2925 (0.3646)	-1.5798 (1.6243)
Constant_Cut4	-0.0624 (0.4123)	-0.0306 (0.9944)	2.2961*** (0.5696)	4.0822*** (1.0838)	1.3328*** (0.3877)	-0.5466 (1.6598)
Constant_Cut5	0.9981** (0.4208)	1.0671 (1.0076)	3.5842*** (0.6329)	5.3624*** (1.1296)	2.7326*** (0.4384)	0.8814 (1.7184)
Constant_Cut6	2.3800*** (0.4911)	2.5078** (1.0475)	4.1966*** (0.6252)	5.9852*** (1.1575)	3.7436*** (0.5267)	1.9035 (1.7382)
Pseudo R-squared	0.02	0.04	0.09	0.09	0.02	0.03
N	672	669	660	654	720	687

*Notes:* Ordered logit regressions run. *NoInt*, *R*1*Int* and *R*2*Int* are dummies that take on the value of 1 if an observation belongs to NoInt, R1Int or R2Int, respectively. *PostRound*1, *PostRound*2 and *PostRound*3 are PostRound dummies. All variables with “*” are interaction terms. Gender is 1 when a participant is male. The rest of the variables are treated as continuous. Standard errors clustered on a group level in parentheses.

*** *p* < 0.01,

** *p* < 0.05,

* *p* < 0.10.

Notably for Denmark and Spain, it is not just the round right after that is positively affected by a first-round intervention. The effect of the intervention in Denmark continues to be positive and statistically significant in PostRound2, not just when compared to PostRound1 of NoInt but also when compared to PostRound2 of NoInt (Wald Test: Column (2) of Table 3, *p* = 0.0040; Column (2) of Table 4, *p* = 0.0110). Unfortunately, in Denmark, PostRound3 under R1Int is no longer statistically significantly different from PostRound3 under NoInt (Wald Test: Column (2) of Table 3, *p* = 0.1180; Column (2) of Table 4, *p* = 0.3160).

We find a first-round intervention to have a much stronger effect on Spaniards than either Danes or Ghanaians. Columns (3) and (4) of Tables 3 and 4 show positive and statistically significant coefficients for PostRound2 and PostRound3 under R1Int. Comparing this to the NoInt coefficients, we find R1Int coefficients to be statistically significantly greater than the NoInt coefficients (Wald Test: PostRound2 Column (3) of Table 3, *p* = 0.0053; PostRound2 Column (4) of Table 4, *p* = 0.0041; PostRound3 Column (3) of Table 3, *p* = 0.0039; PostRound3 Column (4) of Table 4, *p* = 0.0012). Hence, we have the following results:

**Result 2** *An exogenous intervention in the first round increases Danish coordination in the succeeding two rounds*, *Spanish coordination in the succeeding three rounds*, *and Ghanaian coordination in the first succeeding round*.

When an intervention occurs in the second round, we see an increase in coordination and level of coordination among Spaniards but not among Danes and Ghanaians. Tables 3 and 4 show that a second round intervention increases Spanish amount of tokens earned by 21 tokens as a result of increasing the log odds that Spaniards coordinate at a higher level by 1–2. We also see statistically significant coefficients when we regress tokens earned on PostRounds 2 and 3 under R2Int. These coefficients, however, are not statistically significantly different from the coefficients of PostRounds 2 and 3 under NoInt (Wald Test: PostRound2 Column (2) of Table 3, *p* = 0.1061; PostRound2 Column (2) of Table 4, *p* = 0.1480; PostRound3 Column (2) of Table 3, *p* = 0.3906; PostRound3 Column (2) of Table 4, *p* = 0.0806). Hence, we have the following results for R1Int:

**Result 3** *An exogenous intervention in the second round increases the level at which Spaniards coordinate in the succeeding round*. *The same intervention*, *however*, *does not affect coordination among Danes and Ghanaians*.

Looking at Results 2 and 3, we can conjecture that a first-round intervention has a different effect on subsequent coordination than a second-round intervention. Wald tests between R1Int and R2Int coefficients in Denmark reveal that PostRound1 coordination under R1Int is higher than PostRound1 coordination under R2Int (Column (2) of Table 4, *p* = 0.0355). Danish PostRound2 coordination under R1Int is also higher than Danish PostRound2 coordination under R2Int (Column (2) of Table 4, *p* = 0.0405). In Spain, we find that PostRound1 under R1Int is not statistically significantly higher than PostRound1 under R2Int (Wald Test: Column (4) of Table 4, *p* = 0.2170), but PostRound2 and PostRound3 under R1Int is statistically significantly higher than PostRound2 and PostRound3, respectively, under R2Int (Wald Test: PostRound2 Column (4) of Table 4, *p* = 0.0626; PostRound3 Column (4) of Table 4, *p* = 0.0528). In Ghana, we find PostRound1 under R1Int to be statistically significantly higher than PostRound1 under R2Int (Wald Test: Column (6) of Table 4, *p* = 0.0095). Hence, we have the following result:

**Result 4** *A first-round exogenous intervention leads to better coordination for Danes*, *Spaniards and Ghanaians than a second-round exogenous intervention*.

## Discussions and conclusions

We explore how an exogenous intervention affects subsequent individual coordination behavior. The intervention instructs participants in a laboratory experiment to pick the Pareto optimal equilibrium in either the first or second round of a 5-round minimum effort game. We compare individual behavior under these treatments with behavior under a no-intervention baseline. To explore cross-cultural differences in coordination behavior, we undertook identical experiments in Denmark, Spain and Ghana.

We find considerable cross-country variation in coordination behavior with and without intervention across our three countries. Without intervention, we find that Danish students, who belong to a more ethnically, linguistically, and religiously homogeneous population than Spanish and Ghanaian students, to be more coordinated than our Spanish and Ghanaian students. This is in line with the *perceived similarity hypothesis* in the behavioral psychology literature. Individuals who perceive themselves to be more similar are better coordinated [55, 56]. We also find that individuals from countries with higher uncertainty avoidance are more likely to pick lower levels. With almost double the level of uncertainty avoidance measure of Danes, none of our Spanish and Ghanaian participants are able to coordinate at the highest possible effort level in the first round. Many play it safe, coordinating at mid-levels of 3 or 4. As for the subsequent round, we find that the measure for long-term orientation to be correlated with coordination behavior. Spaniards, who have high measures of long-term orientation, are more affected by a positive intervention in the previous round. We see this in the positive increases in coordination after either a first-round or a second-round intervention.

We stress that while we may find cultural measures to correlate in an observable way with coordination behavior, it is important to note that our study shares a limitation with many cross-country studies, namely: the vast number of potential reasons for such cross-country variation makes us unable to draw any meaningful *causal* conclusions.

We do not claim our sample to be representative of the Danish, Spanish and Ghanaian population; and hence, we cannot generalize across the Danish, Spanish and Ghanaian population. The choice to use student samples over representative samples is something we have deliberated at length. The reasons for our choice of participant are as follows. First, since participants in all three countries are students, it is easier to compare baseline coordination behavior across countries since we’re holding educational attainment, profession, and age (to a certain extent at least) constant. Second, like a Danish, Spanish and Ghanaian representative sample, students are products of the society and culture they belong to. Hence, our measures of ethnic, linguistic and religious fractualization, uncertainty avoidance and long-term orientation to describe homogeneity, ambiguity aversion and openness to interventions are descriptive of our student sample. Lastly, when we look at the effects of intervention or when we compare effects across interventions, we do this within (as opposed to across) countries. As such, although our student sample is not representative, it is not the level at which they coordinate that we are interested in but the difference in coordination with and without intervention.

The novelty of our study is to document that an exogenous intervention that instructs individuals to coordinate at the Pareto optimum do indeed influence individual coordination behavior in the subsequent rounds. We find that this is true in all countries when the intervention is done in the first round. It is also true in Spain when the intervention is done in the second round. Coordination decreases the further away the round is from the experience. Nevertheless, Spaniards and Danes are able to coordinate better two and three rounds after a first-round intervention compared to a no-intervention baseline.

The observation that an intervention in the first round leaves a longer lasting mark on the behavior of individuals than an intervention in the second round may indicate the role of such interventions in shaping behavior. We conjecture that individuals who have played the first round of the game without intervention have gained information about how at least one individual in their group behaves. This new information affects their posterior expectations. After a second-round intervention, when they are able to successfully coordinate at the Pareto optimal equilibrium, their expectations are again affected. However, their experience from the first round, when their choices were not dictated, should still play a considerable role in forming their expectations of how the subsequent round will be. Hence, intervening in the first round provides a different initial starting point in individual expectations. When group behavior happens to be what the individual expected, this starting point is reinforced and the effect lasts longer.

Our results suggest interesting implications insofar as coordination among the same group of individuals is repeated. Our Ghanaian and Danish participants are able to coordinate better when they start off on the right foot, i.e., when an intervention that instructs them to coordinate better happens in the first round. This also true for our Spanish participants. However, our Spanish participants are also able to coordinate better even when they do not start off on the right foot, as long as an intervention happens. Starting on the right foot for the Spaniards, however, means that better coordination lasts longer.

Bringing the point back to practical settings that initially motivated our research question, our findings may provide a useful reflection or two for practical coordination challenges. First, instructions and signals to encourage Pareto optimal coordination may be most successful at the start of a new group coordination sequence, e.g. targeting parents at the start of a new school year or targeting colleagues at the start of the year. Second, as coordination deteriorates new interventions, like periodic reminders, may be successful in enhancing coordination in some groups, as indeed common experience would suggest. Again, returning to our first point, our results could indicate that subsequent interventions may benefit from a timing in periods where some degree of expectations reset has occurred.

While our experimental design has something to say about coordination 1 to 3 rounds after an exogenous positive coordination experience, it cannot really say anything about how coordination would be in the very long run, i.e., 20 to 30 rounds after. How slow does coordination decline after experience and how long will it take for coordination after experience to catch up with coordination without experience? Will the effect of the experience be more positive when the experience lasts for more than a round? What happens when there are intermittent experiences? These are questions that our current paper cannot answer, but are definitely worth doing further research on.

## Supporting information

S1 File Experiment instructions, questionnaire and consent form. Experiment instructions for the minimum effort game with and without intervention. Since rounds 3, 4 and 5 are rounds with no intervention, the instructions for these rounds are exactly the same as the instructions for round 2 without intervention. Instruction for round 1 with intervention is labeled as “EXPERIMENT INSTRUCTIONS (ME-R1IG)”, where *ME* stands for “Minimum Effort”, *R1* stands for “Round 1”, *I* stands for “Intervention” (*N* stands for “No Intervention”), and *G* stands for “Ghana”. Consent forms were signed at the start of the experiment while questionnaires and payment forms were filled out at the end.
(PDF)

S1 Appendix Summary statistics: Demographics. (PDF)

S2 Appendix Statistical test: Demographics. (PDF)

S3 Appendix Results for t-tests. (PDF)
