# Supplementary material for: One-shot exogenous interventions increase subsequent coordination in Denmark, Spain and Ghana
Source: PLoS One. 2017 Nov 16;12(11):e0187840. doi: 10.1371/journal.pone.0187840 (PMC5690427; doi:10.1371/journal.pone.0187840)
Supplement: S1 File — Experiment instructions for the minimum effort game with and without intervention. Since rounds 3, 4 and 5 are rounds with no intervention, the instructions for these rounds are exactly the same as the instructions for round 2 without intervention. Instruction for round 1 with intervention is labeled as “EXPERIMENT INSTRUCTIONS (ME-R1IG)”, where ME stands for “Minimum Effort”, R1 stands for “Round 1”, I stands for “Intervention” (N stands for “No Intervention”), and G stands for “Ghana”. Consent forms were signed at the start of the experiment while questionnaires and payment forms were filled out at the end. (PDF) [file pone.0187840.s001.pdf]

Consent to Participate in a Research Project:
"Decision-making experiment"

The purpose of our research project is to study the economics of decision-making. We are asking you to participate in this project because we believe that observing how real people make decisions is informative about decision-making in the real world. The results of this project will help broaden society's understanding of economic decision-making under different conditions.

Project Description - Activities and Time Commitment: If you participate, you will make independent decisions on your own, and maybe interact with other participants. Each activity will involve making concrete decisions or choices.

Experimental Instructions, which will be explained to you in detail, gives a detailed description of each activity.

Benefits and Risks: We believe there are no direct benefits to you for participating in our research project, except for the modest monetary incentives we explain below. However, the results of this project might help to broaden society's understanding of economic decision-making under different conditions. We believe there is no risk to you in participating in this project. If, however, you are uncomfortable or stressed by participating in any of the activities, you can take a break, or withdraw from the project altogether.

Confidentiality and Privacy: During this research project, we will keep all data in a secure location. Only we will have access to the data, although legally authorized agencies, including the University of Copenhagen's Responsible Conduct of Research Committee, have the right to review research records.

Each participant will be identified by a Subject ID number only and no information collected can be related to your name and person. Only the information on individual decisions and a brief follow-up questionnaire will be recorded. When we report the results of our research project, we will and can not use your name or any other personally identifying information, as the experiment is entirely anonymous. If you would like a summary of the findings from our final report, please contact us using the email listed near the end of this consent form.

Voluntary Participation: Participation in this research project is voluntary. You can choose freely to participate or not to participate. In addition, at any point during this project, you can withdraw your permission without any penalty of loss of benefits.

Compensation:
You will be given 3 EUR for your participation. Additionally, some of the activities have a monetary incentive. Participants will be paid in private and their earning will not be revealed to other participants. You will be responsible for declaring your earnings in this experiment as part of your taxable income.

Questions: If you have any questions about this project, please contact Anna via email:
Anna Abatayo, anna.abatayo@gmail.com

If you agree to participate in this project, please sign the signature portion of this consent form.

Signature(s) for Consent:

I agree to participate in the research project entitled, **"Decision-making experiment"**

I understand that I can change my mind about participating in this project, at any time, by notifying the researcher.

Your Name (Print): ____

Your Signature/Thumbmark: ____

Date: _________________________________

Subject ID Number: _________
Group ID Number: __________

EXPERIMENT INSTRUCTIONS (ME-R1IG)

Introduction

Welcome to the experiment.

For showing up to today's session, you will be paid 3 GHS. You will have a chance to receive additional money as the result of the outcomes in each part of the experiment. All your earnings will be in experimental dollars (E$). At the end of the experiment, we will sum up all the E$ you earned and we will pay you in cash an amount equal to 0.40 GDS for every E$ you earn.

You are not allowed to communicate with other participants during the experiment. Should you have any questions, please raise your hand and ask the instructor. If you violate this rule, we will have to exclude you from the experiment and from all payments.

On the upper left side of your table is your **Subject ID Number**. This will be your ID number for the duration of the experiment.

You will also find on your table a calculator, a pen and a piece of blank paper for your personal notes. You are free to use the calculator, the pen, and the blank paper anytime during the experiment.

Are there any questions?

This experiment is composed of 5 rounds. In each round, you have the possibility of earning E$. At the end of the experiment, we will sum up all your earnings in each round.

Are there any questions?

Decision and payoffs

In this round, you have been randomly assigned to a group of 4. Your **Group ID Number** can be found at the upper left side of this page, below your **Subject ID Number**.

To earn E$, you and the people in your group will be selecting a number from 1 to 7. Your earnings depend on the number of tokens you have. In turn, the number of tokens you have depends on two things: *i*) the number you have chosen and *ii*) the minimum number chosen by you and the other members of your group.

The table below shows the number of tokens you can accumulate based on these two things. Each token is equivalent to 0.06 E$. Everyone has the same table in their instructions.

Minimum Choice in Group

Your Own Choice	1	2	3	4	5	6	7
**1**	70						
**2**	60	80					
**3**	50	70	90				
**4**	40	60	80	100			
**5**	30	50	70	90	110		
**6**	20	40	60	80	100	120	
**7**	10	30	50	70	90	110	130

For example, if you choose 5, and the minimum choice in your group is 4, then you will accumulate 90 tokens.

Minimum Choice in Group

Your Own Choice	1	2	3	4	5	6	7
**1**	70						
**2**	60	80					
**3**	50	70	90				
**4**	40	60	80	100			
**5**	30	50	70	90	110		
**6**	20	40	60	80	100	120	
**7**	10	30	50	70	90	110	130

Are there any questions?

Entering Decisions

When you make your choice, take one of the small pieces of paper labeled “EXPERIMENT CARD (ME)” and write your chosen number next to “My Chosen Number”. You must also write this number in the first column (Column A) of “Round No. 1” on the RECORD SHEET. This means that you are writing your decision down in 2 places: the EXPERIMENT CARD (ME) that the instructor will collect from you and the RECORD SHEET that you hang on to.

The instructor will collect the EXPERIMENT CARD (ME) from everyone and will identify the smallest number picked in the group. The instructor will announce this number publicly by calling out the Group Number first and then the group's minimum amount. Once the instructor announces your group's minimum amount, please write down this amount under "Minimum Number Chosen in Group" on the RECORD SHEET. You can compute your accumulated tokens by using the table above. Write your accumulated tokens down in Column C of the RECORD SHEET. Following the above example, you should write down "4" under Column B and "90" under Column C of the RECORD SHEET.

When making your decisions for this round, you MUST write "7" on your "EXPERIMENT CARD (ME)" and on your RECORD SHEET.

Are there any questions?

You may now answer the review questions below. When you are finished, please raise your hand and wait for the instructor to check your answers.

Review

1. How many people will there be in a group? ____
2. If you chose 6 and the rest of your group chose 3, 4, and 7 how many tokens will you accumulate?____
3. If you chose 4 and the rest of your group chose 5, 6 and 6, how many tokens will you accumulate? ____

Subject ID Number: _________
Group ID Number: __________

EXPERIMENT INSTRUCTIONS (ME-R1NG)

Introduction

Welcome to the experiment.

For showing up to today's session, you will be paid 3 GHS. You will have a chance to receive additional money as the result of the outcomes in each part of the experiment. All your earnings will be in experimental dollars (E$). At the end of the experiment, we will sum up all the E$ you earned and we will pay you in cash an amount equal to 0.40 GDS for every E$ you earn.

You are not allowed to communicate with other participants during the experiment. Should you have any questions, please raise your hand and ask the instructor. If you violate this rule, we will have to exclude you from the experiment and from all payments.

On the upper left side of your table is your **Subject ID Number**. This will be your ID number for the duration of the experiment.

You will also find on your table a calculator, a pen and a piece of blank paper for your personal notes. You are free to use the calculator, the pen, and the blank paper anytime during the experiment.

Are there any questions?

This experiment is composed of 5 rounds. In each round, you have the possibility of earning E$. At the end of the experiment, we will sum up all your earnings in each round.

Are there any questions?

Decision and payoffs

In this round, you have been randomly assigned to a group of 4. Your **Group ID Number** can be found at the upper left side of this page, below your **Subject ID Number**.

To earn E$, you and the people in your group will be selecting a number from 1 to 7. Your earnings depend on the number of tokens you have. In turn, the number of tokens you have depends on two things: *i*) the number you have chosen and *ii*) the minimum number chosen by you and the other members of your group.

The table below shows the number of tokens you can accumulate based on these two things. Each token is equivalent to 0.06 E$. Everyone has the same table in their instructions.

Minimum Choice in Group

Your Own Choice		1	2	3	4	5	6	7
	1	70						
	2	60	80					
	3	50	70	90				
	4	40	60	80	100			
	5	30	50	70	90	110		
	6	20	40	60	80	100	120	
	7	10	30	50	70	90	110	130

For example, if you choose 5, and the minimum choice in your group is 4, then you will accumulate 90 tokens.

Minimum Choice in Group

Your Own Choice		1	2	3	4	5	6	7
	1	70						
	2	60	80					
	3	50	70	90				
	4	40	60	80	100			
	5	30	50	70	90	110		
	6	20	40	60	80	100	120	
	7	10	30	50	70	90	110	130

Are there any questions?

Entering Decisions

When you make your choice, take one of the small pieces of paper labeled "EXPERIMENT CARD (ME)" and write your chosen number next to "My Chosen Number". You must also write this number in the first column (Column A) of "Round No. 1" on the RECORD SHEET. This means that you are writing your decision down in 2 places: the EXPERIMENT CARD (ME) that the instructor will collect from you and the RECORD SHEET that you hang on to.

The instructor will collect the EXPERIMENT CARD (ME) from everyone and will identify the smallest number picked in the group. The instructor will announce this number publicly by calling out the Group Number first and then the group's minimum amount. Once the instructor announces your group's minimum amount, please write down this amount under "Minimum Number Chosen in Group" on the RECORD SHEET. You can compute your accumulated tokens by using the table above. Write your accumulated tokens down in Column C of the RECORD SHEET. Following the above example, you should write down "4" under Column B and "90" under Column C of the RECORD SHEET.

Are there any questions?

You may now answer the review questions below. When you are finished, please raise your hand and wait for the instructor to check your answers.

Review

1. How many people will there be in a group? ____
2. If you chose 6 and the rest of your group chose 3, 4, and 7 how many tokens will you accumulate?____
3. If you chose 4 and the rest of your group chose 5, 6 and 6, how many tokens will you accumulate? ____

EXPERIMENT INSTRUCTIONS (ME-R2IG)

You will remain in your previous group of 4 and choose a number between 1 and 7. This time, please write your choice, your group's minimum choice, and your accumulated tokens under "**Round No. 2**" of the RECORD SHEET.

When making your decisions for this round, you **MUST** write "7" on your "EXPERIMENT CARD (ME)" and on your RECORD SHEET.

Everything else is the same as before.

Are there any questions?

EXPERIMENT INSTRUCTIONS (ME-R2NG)

You will remain in your previous group of 4 and choose a number between 1 and 7. This time, please write your choice, your group's minimum choice, and your accumulated tokens under "**Round No. 2**" of the RECORD SHEET.

Everything else is the same as before.

Are there any questions?

QUESTIONNAIRE (Ghana)

Thank you for participating in our decision-making experiment. We are now preparing your earnings from our experiment. While we do that, we would like you to answer a questionnaire. Your answers in the questionnaire will not affect your payoffs in any way.

Please fill in the blanks or shade your answer. Note that all answers are anonymous as we only relate them to your Subject ID.

Subject ID Number: _________

Age: __________

Gender:
- o Male
- o Female

Are you a student
- o Yes
- o No

Field of Study: _________________________________

Highest Educational Attainment: ___________

Marital Status
- o Single
- o Co-habiting
- o Married
- o Widowed

Number of Children: ___________

Do you believe that individuals in Denmark and Spain had the same experiment you had?

☐ Yes

☐ No

Are you generally a person who is fully prepared to take risks or do you try to avoid taking risks? Please tick a box on the scale below.

Not at all willing to take risks<br>1									Very willing to take risks<br>10
☐	☐	☐	☐	☐	☐	☐	☐	☐	☐

Thinking about people from **Denmark**, how much do you agree with the following statements:

	Strongly agree	Kind of agree	Kind of disagree	Strongly disagree	I don't know
I like the **Danes**	☐	☐	☐	☐	☐
I think **Danes** can be trusted	☐	☐	☐	☐	☐
I think **Danes** are ***not*** very cooperative	☐	☐	☐	☐	☐
I think **Danes** care for nature	☐	☐	☐	☐	☐
I think **Danes** will ***not*** protect the habitats of the migratory birds	☐	☐	☐	☐	☐
I think **Danes** are quite wealthy	☐	☐	☐	☐	☐
I think **Danes** do ***not*** care what happens to my country and my people	☐	☐	☐	☐	☐

Thinking about people from **Spain**, how much do you agree with the following statements:

	Strongly agree	Kind of agree	Kind of disagree	Strongly disagree	I don't know
I like the **Spanish**	☐	☐	☐	☐	☐
I think the **Spanish** can be trusted	☐	☐	☐	☐	☐
I think the **Spanish** are ***not*** very cooperative	☐	☐	☐	☐	☐
I think the **Spanish** care for nature	☐	☐	☐	☐	☐
I think the **Spanish** will ***not*** protect habitats	☐	☐	☐	☐	☐
I think the **Spanish** are quite wealthy	☐	☐	☐	☐	☐
I think the **Spanish** do ***not*** care what happens to my country and my people	☐	☐	☐	☐	☐

Thinking about people from **Ghana**, how much do you agree with the following statements:

	Strongly agree	Kind of agree	Kind of disagree	Strongly disagree	I don't know
I like the **Ghanaians**	☐	☐	☐	☐	☐
I think the **Ghanaians** can be trusted	☐	☐	☐	☐	☐
I think the **Ghanaians** are ***not*** very cooperative	☐	☐	☐	☐	☐
I think the **Ghanaians** care for nature	☐	☐	☐	☐	☐
I think the **Ghanaians** will ***not*** protect habitats	☐	☐	☐	☐	☐
I think the **Ghanaians** are quite wealthy	☐	☐	☐	☐	☐
I think the **Ghanaians** do ***not*** care what happens to my country and my people	☐	☐	☐	☐	☐

Thank you.

The experiment is over. We will now be calling you one by one to pay you your show-up fee. We will also need to collect your signatures as proof that we paid you money as a participant of this experiment. We will NOT be keeping this form with your signature. It will immediately go to the accounting office of the research grant that is funding this experiment.

When your Subject ID Number is called, please bring your answered questionnaire and your pens.

SUBJECT ID NUMBER:________

DATE: ___________________

TIME:____________________

PAYMENT FORM

- Show-up Fee: ______________________
- Earnings: ___________________________

TOTAL EARNINGS*: ____________________

*Show-up Fee + Money Kept + PG Earnings + ME Earnings

I received my show-up fee and my earnings from the experiment.

____

Signature or Thumb mark
