# Supplementary material for: One-shot exogenous interventions increase subsequent coordination in Denmark, Spain and Ghana
Source: PLoS One. 2017 Nov 16;12(11):e0187840. doi: 10.1371/journal.pone.0187840 (PMC5690427; doi:10.1371/journal.pone.0187840)
Supplement: S1 Appendix — (PDF) [file pone.0187840.s002.pdf]

S1 Appendix. Summary Statistics: Demographics

	Denmark								
	NoInt			R1Int			R2Int		
	Obs.	Mean	Std. Dev.	Obs.	Mean	Std. Dev.	Obs.	Mean	Std. Dev.
Age	72	23.88	3.06	80	24.81	5.87	72	23.69	3.86
Gender	72	0.55	0.50	80	0.43	0.49	72	0.43	0.50
Civil Status	71	1.41	0.57	79	1.51	0.63	72	1.42	0.55
Nos. Children	71	0.07	0.31	80	0.16	0.60	72	0.07	0.39
	Spain								
	NoInt			R1Int			R2Int		
	Obs.	Mean	Std. Dev.	Obs.	Mean	Std. Dev.	Obs.	Mean	Std. Dev.
Age	72	20.65	2.81	76	21.49	5.12	72	20.88	1.99
Gender	72	0.32	0.47	76	0.37	0.49	72	0.32	0.47
Civil Status	72	1.07	0.25	76	1.18	0.48	72	1.11	0.36
Nos. Children	72	0	0	76	0.01	0.11	72	0	0
	Ghana								
	NoInt			R1Int			R2Int		
	Obs.	Mean	Std. Dev.	Obs.	Mean	Std. Dev.	Obs.	Mean	Std. Dev.
Age	84	22.37	1.89	83	22.30	1.70	72	22.75	1.70
Gender	84	0.68	0.47	82	0.78	0.42	72	0.79	0.41
Civil Status	84	1.05	0.31	83	1	0	72	1	0
Nos. Children	84	0.05	0.31	83	0	0	72	0	0

Notes: Age is measured in years. Gender is a binary variable which takes on the value of 1 if male and 0 if female. Civil Status is equal to 1 if single, 2 if co-habiting, 3 if married, and 4 if widowed.
