# Supplementary material for: One-shot exogenous interventions increase subsequent coordination in Denmark, Spain and Ghana
Source: PLoS One. 2017 Nov 16;12(11):e0187840. doi: 10.1371/journal.pone.0187840 (PMC5690427; doi:10.1371/journal.pone.0187840)
Supplement: S2 Appendix — (PDF) [file pone.0187840.s003.pdf]

S2 Appendix. Statistical Tests: Demographics

Two-Sided t-Tests on Participant Demographics

	Gender		
	NoInt vs. R1Int	NoInt vs. R2Int	R1Int vs. R2Int
Denmark	0.1847	0.1526	0.9453
Spain	1	0.5341	0.5341
Ghana	0.1138	0.1414	0.8671
	Age		
	NoInt vs. R1Int	NoInt vs. R2Int	R1Int vs. R2Int
Denmark	0.9867	0.2494	0.2663
Spain	1	0.3321	0.3321
Ghana	0.1899	0.1592	1

Notes: Tests where done on an individual level.

Two-Sided Kolmogorov-Smirnov Tests on Participant Demographics

	Age		
	NoInt vs. R1Int	NoInt vs. R2Int	R1Int vs. R2Int
Denmark	0.995	0.432	0.384
Spain	0.131	0.992	0.427
Ghana	0.151	0.985	0.425
	Civil Status		
	NoInt vs. R1Int	NoInt vs. R2Int	R1Int vs. R2Int
Denmark	1	0.998	1
Spain	1	0.935	0.999
Ghana	1	1	1

Notes: Tests where done on an individual level.
