# Supplementary material for: One-shot exogenous interventions increase subsequent coordination in Denmark, Spain and Ghana
Source: PLoS One. 2017 Nov 16;12(11):e0187840. doi: 10.1371/journal.pone.0187840 (PMC5690427; doi:10.1371/journal.pone.0187840)
Supplement: S3 Appendix — (PDF) [file pone.0187840.s004.pdf]

S3 Appendix. Results for t-Tests

Table 1: t-Test Results Between Treatments

	PostRound1								
	NoInt vs. R1Int			NoInt vs. R2Int			R1Int vs. R2Int		
	$p < 0$	$p = 0$	$p > 0$	$p < 0$	$p = 0$	$p > 0$	$p < 0$	$p = 0$	$p > 0$
Denmark	0.0063	0.0126	0.9937	0.1227	0.2453	0.8773	0.9147	0.1706	0.0853
Spain	0.0002	0.0004	0.9998	0.0057	0.0114	0.9943	0.8807	0.2386	0.1193
Ghana	0.0005	0.001	0.001	0.6365	0.7271	0.3635	0.9995	0.001	0.0005
	PostRound2								
	NoInt vs. R1Int			NoInt vs. R2Int			R1Int vs. R2Int		
	$p < 0$	$p = 0$	$p > 0$	$p < 0$	$p = 0$	$p > 0$	$p < 0$	$p = 0$	$p > 0$
Denmark	0.0247	0.0495	0.9753	0.3545	0.709	0.6455	0.6982	0.6035	0.3018
Spain	0.0007	0.0015	0.9993	0.2429	0.4858	0.7571	0.6033	0.7934	0.3967
Ghana	0.352	0.7043	0.6479	0.7316	0.5368	0.2684	0.853	0.2941	0.147
	PostRound3								
	NoInt vs. R1Int			NoInt vs. R2Int			R1Int vs. R2Int		
	$p < 0$	$p = 0$	$p > 0$	$p < 0$	$p = 0$	$p > 0$	$p < 0$	$p = 0$	$p > 0$
Denmark	0.0487	0.0974	0.9513	0.4266	0.8532	0.5734	0.648	0.7039	0.352
Spain	0.0001	0.0001	0.9999	0.0153	0.0306	0.9847	0.5489	0.9022	0.4511
Ghana	0.3336	0.6671	0.6664	0.6153	0.7695	0.3847	0.8746	0.2509	0.1254

Table 2: t-Test Results Between Rounds

	PostRound1 vs. PostRound2								
		NoInt			R1Int			R2Int	
	p < 0	p = 0	p > 0	p < 0	p = 0	p > 0	p < 0	p = 0	p > 0
Denmark	0.6336	0.7328	0.3664	0.7323	0.5355	0.2677	0.8156	0.3687	0.1844
Spain	0.6837	0.6327	0.3163	0.9176	0.1649	0.0824	0.9818	0.0363	0.0182
Ghana	0.3769	0.7538	0.6231	0.9945	0.0111	0.0055	0.5	1	0.5
	PostRound1 vs. PostRound3								
		NoInt			R1Int			R2Int	
	p < 0	p = 0	p > 0	p < 0	p = 0	p > 0	p < 0	p = 0	p > 0
Denmark	0.8139	0.3723	0.1861	0.9502	0.0996	0.0498	0.9543	0.0914	0.0457
Spain	0.9987	0.0027	0.0013	0.9905	0.019	0.0095	0.9997	0.0006	0.0003
Ghana	0.7861	0.4278	0.2139	0.9979	0.0043	0.0021	0.6221	0.7558	0.3779
	PostRound2 vs. PostRound3								
		NoInt			R1Int			R2Int	
	p < 0	p = 0	p > 0	p < 0	p = 0	p > 0	p < 0	p = 0	p > 0
Denmark	0.8892	0.2216	0.1108	0.8768	0.2464	0.1232	0.8318	0.3363	0.1682
Spain	0.9995	0.0011	0.0005	0.9197	0.1606	0.0803	0.9199	0.1602	0.0801
Ghana	0.6722	0.6556	0.3278	0.6987	0.6025	0.3013	0.6174	0.7653	0.3826
